# Decarboxylation mechanisms of C4 photosynthesis in *Saccharum* spp.: increased PEPCK activity under water-limiting conditions

**DOI:** 10.1186/s12870-019-1745-7

**Published:** 2019-04-16

**Authors:** Viviane Cacefo, Alessandra Ferreira Ribas, Rafael Rebes Zilliani, Daniel Moreira Neris, Douglas Silva Domingues, Adriana Lima Moro, Luiz Gonzaga Esteves Vieira

**Affiliations:** 1 Centro de Estudos em Ecofisiologia Vegetal do Oeste Paulista (CEVOP), Universidade do Oeste Paulista (UNOESTE), Rodovia Raposo Tavares, Km 572, CEP, Presidente Prudente, SP 19067-175 Brazil; 2 Agronomy Graduate Program, Universidade do Oeste Paulista (UNOESTE), Rodovia Raposo Tavares, Km 572, CEP, Presidente Prudente, SP 19067-175 Brazil; 3 Departamento de Botânica, Instituto de Biociências de Rio Claro, Universidade Estadual Paulista (UNESP), Avenida 24-A, 1515, CEP, Rio Claro, SP 13506-900 Brazil

**Keywords:** *Saccharum* spp. drought stress. C4 photosynthesis. Decarboxylation mechanisms. Gene regulation

## Abstract

**Background:**

C4 plants have been classified into three subtypes based on the enzymes used to decarboxylate C4 acids in the bundle sheath cells (NADP-ME, NAD-ME and PEPCK pathways). Evidences indicate that, depending on environmental factors, C4 plants may exhibit a certain degree of flexibility in the use of the decarboxylation mechanisms. In this context, the objective was to extend the knowledge on the degree of flexibility between the pathways of decarboxylation in sugarcane, a NADP-ME species, at different levels of water deficit.

**Results:**

An experiment was carried out with two cultivars - RB92579 (tolerant to water deficit) and SP80–3280 (susceptible to water deficit) subjected to moderate level (− 1.5 to − 1.8 MPa), severe level (below − 2.0 MPa) and recovery (48 h after rehydration) and changes in the activities of the enzymes involved in the three C4 mechanisms and in gene expression were investigated. Our results showed that sugarcane uses the PEPCK pathway as a decarboxylation mechanism in addition to the NADP-ME, which was more evident under water deficit conditions for both cultivars.

**Conclusions:**

The results obtained here, show that sugarcane increases the use of the PEPCK pathway as a decarboxylation mechanism, in addition to the NADP-ME pathway, under conditions of water deficit, particularly in the tolerant cultivar.

**Electronic supplementary material:**

The online version of this article (10.1186/s12870-019-1745-7) contains supplementary material, which is available to authorized users.

## Background

Sugarcane, like other species classified as having C4 photosynthetic pathways, developed anatomical and physiological adaptations to optimize CO_2_ fixation for carbohydrate synthesis [1]. Plants of the C4 photosynthetic pathway are grouped in three biochemical subtypes, according to whether they contained high levels of NADP-malic enzyme (NADP-ME, EC 1.1.1.40), phosphoenolpyruvate carboxykinase (PEPCK, EC 4.1.1.49) or NAD-malic enzyme (NAD-ME, EC 1.1.1.39) for the decarboxylation of the C4 acids. The decarboxylation in the bundle sheath cells can occur in distinct cellular compartments, since NADP-ME occurs in chloroplasts, NAD-ME in mitochondria, whereas PEPCK exhibits cytosolic activity [2]. Currently, sugarcane is classified as a NADP-ME subtype [3].

CO_2_ is initially fixed in the mesophyll cells by phosphoenolpyruvate carboxylase (PEPC), a common enzyme in all three in C4 subtypes (Additional file 1: Figure S1). The product formed is oxaloacetate (OAA), which can be either reduced to malate by the NADP-dependent malate dehydrogenase (NADP-MDH) or converted to aspartate by the aspartate aminotransferase (AspAT). In the NADP-ME subtype, the malate is transported to the bundle sheath cells where it is converted to pyruvate, releasing CO_2_. Next, pyruvate returns to the mesophyll cells and the orthophosphate pyruvate dikinase (PPDK) catalyzes the formation of phosphoenolpyruvate (PEP). In the NAD-ME and PEPCK subtypes, aspartate is transported to the bundle sheath cells and converted back into OAA in the mitochondria and cytosol, respectively. In the NAD-ME subtype, OAA is reduced to malate by NADP-MDH, and then NAD-ME catalyzes the oxidative conversion of malate into pyruvate, releasing CO_2_ into the mitochondria [4, 5].

In the PEPCK subtype, most of the OAA is converted into PEP by the enzyme PEPCK and CO_2_ is released in the cytosol of the bundle sheath cells. The NAD-ME enzyme provides the reducing equivalents as NADH for the generation of ATP for the PEPCK reaction. The resulting pyruvate can be converted to alanine by the enzyme alanine aminotransferase (AlaAT), which returns to the mesophyll cells (Additional file 1: Figure S1) [4, 5]. Whether PEPCK is an independent biochemical subtype or whether it is essentially similar to NAD-ME or NADP-ME species remains unresolved.

Several pieces of molecular, biochemical and physiological information indicate that C4 plants of the NADP-ME subtype, such as sugarcane, maize and sorghum, could exhibit a certain level of flexibility in the use of the three decarboxylation subtypes (NADP-ME, NAD-ME and PEPCK) and that these subtypes may not be genetically determined in a rigid manner [5]. Flexibility, in this context, can be defined as the variation in the flux of more than one C4 acid decarboxylation pathway in the same plant depending on the environmental conditions.

Indeed, the evidence of mixed pathways of decarboxylation was detected very early, in which approximately 25% of the C^14^-labeled CO_2_ was incorporated into aspartate in species classified as NADP-ME subtype [6]. In addition, in experiments with maize, significant amounts of labeled aspartate were produced and isolated bundle sheath cells were able to use aspartate and oxoglutarate to generate CO_2_ to maintain photosynthesis [7]. High PEPCK expression levels were detected in bundle sheath cells of maize plants [8]. PEPCK activity was verified in bundle sheath cells in maize, *Echinochloa colona, E. crus-galli*, *Digitaria sanguinalis* and *Paspalum notatum*, categorized as NADP-ME species, but not observed in NAD-ME subtype species [9]. Specifically, in sugarcane, a study using the SAGE (Serial Analysis of Gene Expression) technique showed that *PEPCK* transcript was more abundant than that of NADP-ME in bundle sheath cells [10]. Recently, was reported the activity of the three decarboxylases (NADP-ME, NAD-ME and PEPCK) in sugarcane plants and that shading caused increases in the decarboxylation through PEPCK [11]. This flexibility in the use of enzymes of different C4 subtypes has direct consequences on energy requirements for photosynthesis and, consequently, for the acclimation to fluctuating environmental conditions [5, 11–13].

The C4 photosynthetic pathways can be differentially controlled depending on different factors such as environmental conditions and plant developmental stages. In a study using three different C4 grasses, *Paspalum dilatatum* (NADP-ME subtype), *Cynodon dactylon* (NAD-ME) and *Zoysia japonica* (PEPCK), it was demonstrated that moderate leaf dehydration had a species-specific effect on the C4 decarboxylases, mostly by increasing the PEPCK activity in all three species [14]. In view of these studies, it is apparent that both NADP-ME and PEPCK enzymes are present in the bundle sheath cells in NADP-ME species, using malate and/or aspartate as the C4 translocated acid to generate CO_2_ to support photosynthesis [12, 15].

Water deficit is one of the main constraints that restricts the capacity of sugarcane plants to perform important biochemical functions during crop development. One of the first responses to water limitation is the reduction of the photosynthetic rate due to the stomatal closure and decline in CO_2_ assimilation, which in turn alters the activity of enzymes such as Rubisco, PEPC, PPDK, NADP-ME and others [16]. Under water deficit, non-stomatal limitations, such as reduced substrate supply to carboxylases, may cause sufficient metabolic inhibition in the C4 grasses to reduce CO_2_ assimilation by approximately 40% [17]. Additionally, there is evidence that abiotic disturbances, such as drought, affects not only the activities of the enzymes associated with C4 photosynthesis but also the transcriptional levels of the genes encoding these enzymes [18]. These are all regulatory mechanisms that may alter in response to abiotic stresses but that are still undefined at the molecular level in sugarcane. Thus, the flexibility of the C4 decarboxylation mechanism to maintain photosynthetic efficiency could be an important factor in plants grown under environmental stressful conditions, such as water deficit [5, 12].

Therefore, the objective of this work was to study the enzymes involved in the three decarboxylation C4 subtypes (NAD-ME, NADP-ME e PEPCK) in sugarcane plants to verify the possible flexibility between the pathways in this important NADP-ME crop species subjected to different water deficit levels. For this, we (a) characterized two sugarcane cultivars with distinct responses to water deficit conditions (RB92579, tolerant, and SP80–3280, susceptible to water deficit), (b) established comparative analyses of the activities of the enzymes that typify the three subtypes and (c) measured the changes in the transcripts of genes encoding enzymes involved in the C4 acid decarboxylation mechanisms.

## Results

### Water status, leaf gas exchange and biomass analyses

**Fig. 1:**
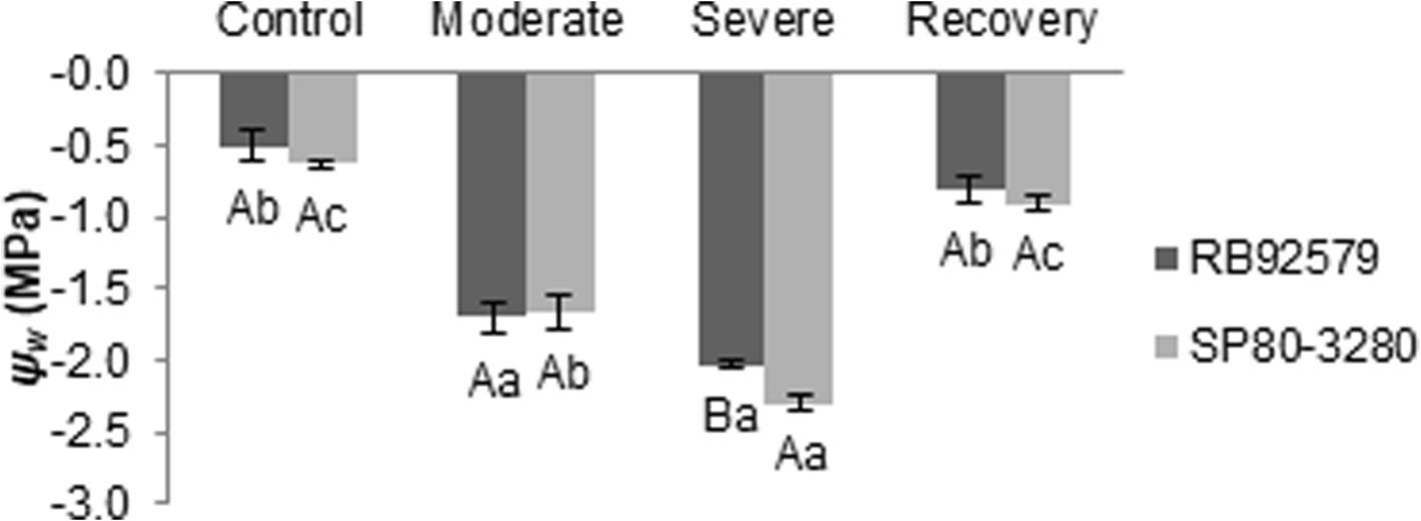
Leaf water potential (*ψ*_*w*_) for tolerant (RB92579) and susceptible (SP80–3280) sugarcane plants under different water deficit regimes. Different upper case and lower letters indicate statistical differences between cultivars in each treatment and between water deficit treatments (control, moderate, severe and recovery) for each cultivar, respectively, according to Tukey test (*P* < 0.05). Values presented as mean ± SE (*n* = 3)

Water deficit was assessed daily by measuring the leaf water potential values (*ψ*_*w*_). The plants reached the pre-established moderate water deficit level (*ψ*_*w*_ = − 1.5 to − 1.8 MPa) three days after the onset of the water deprivation, while the severe condition (*ψ*_*w*_ = below − 2.0 MPa) occurred nine days after attaining the previous water deficit level. We only observed differences in the water potential values between the susceptible and tolerant plants at the end of the experimental period (12 days) when the plants reached the maximum applied water deficit level. SP80–3280 plants showed a reduction in *ψ*_*w*_ (− 2.3 MPa) compared with RB92579 (− 2.0 MPa), confirming its lower tolerance to water deprivation. After re-watering, both cultivars recovered the *ψ*_*w*_ values recorded in plants under normal irrigation conditions (Fig. 1).

**Fig. 2:**
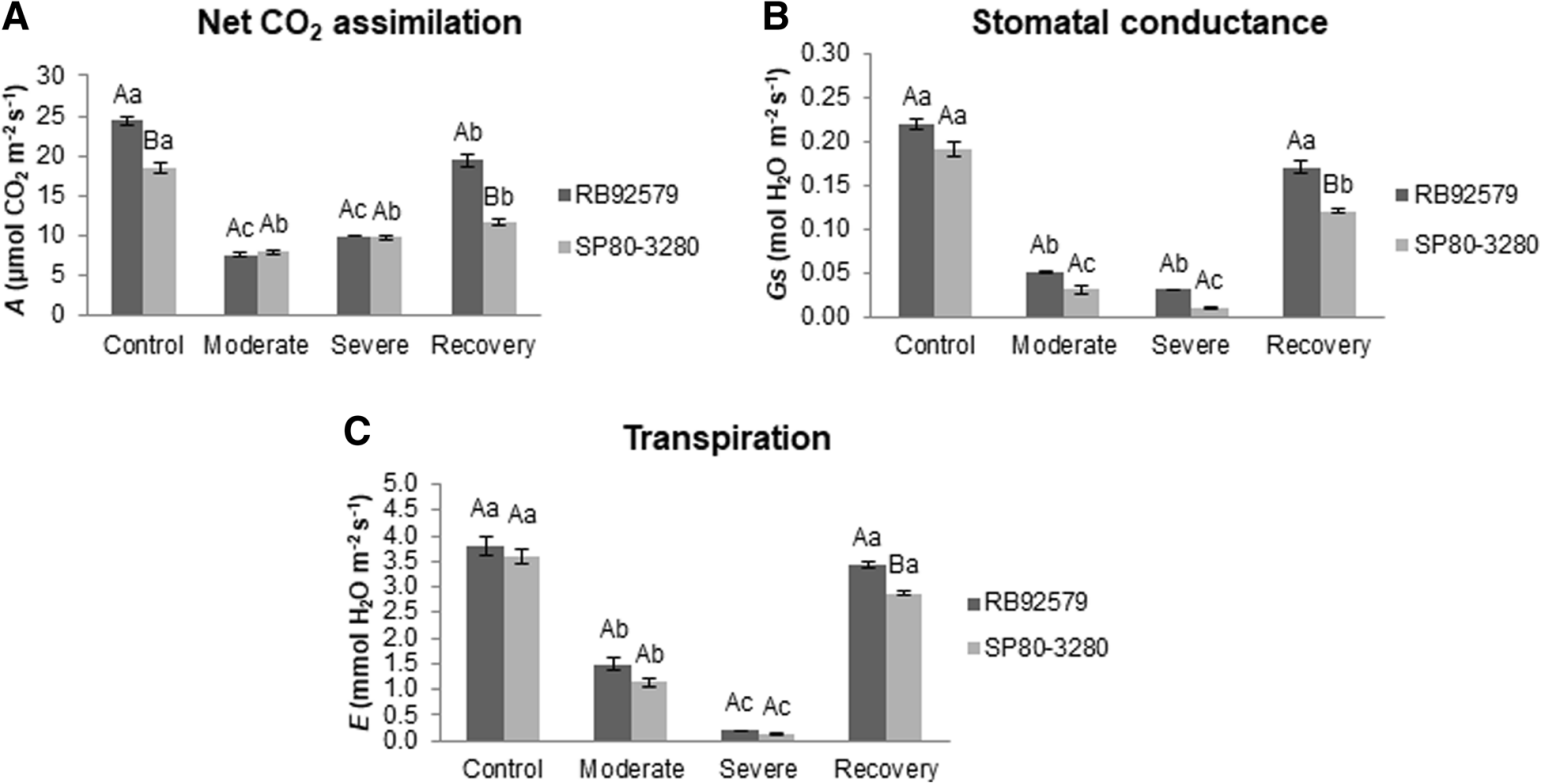
Leaf gas exchange analysis in tolerant (RB92579) and susceptible (SP80–3280) sugarcane plants under different water deficit regimes. **a** - Net CO_2_ assimilation (*A*, μmol CO_2_ m^− 2^ s^− 1^); **b** - stomatal conductance (*Gs*, mol H_2_O m^− 2^ s^− 1^); C - transpiration (*E*, mmol H_2_O m^− 2^ s^− 1^). Different upper case and lower letters indicate statistical differences between cultivars in each treatment and between water deficit treatments (control, moderate, severe and recovery) for each cultivar, respectively, according to Tukey test (*P* < 0.05). Values presented as mean ± SE (*n* = 3)

The water deficit reduced the leaf gas exchange in both sugarcane cultivars (Fig. 2). The tolerant plants (RB92579) showed higher CO_2_ assimilation rate (*A*) under normal water supply condition. However, when subjected to water deficit, RB92579 and SP80–3280 showed reduced CO_2_ assimilation of 7.56 and 7.92 μmol CO_2_ m^− 2^ s^− 1^, respectively, but without significant differences between them and between the moderate and severe water deficit levels within cultivars (Fig. 2a). The susceptible plants (SP80–3280) showed low photosynthetic activity (around 50% of the irrigated control) in the recovery. On the other hand, the CO_2_ assimilation also declined in the tolerant plants after water deprivation, but the photosynthesis level was reestablished at similar levels as the irrigated control plants at the beginning of the experiment (Fig. 2a).

A decline in stomatal conductance (*Gs*) was observed until the end of the water deficit treatment in both cultivars (Fig. 2b). This decline was observed in plants under the moderate water deficit, in which these values ranged from 0.05 to 0.03 mol H_2_O m^− 2^ s^− 1^ on RB92579 and SP80–3280, respectively (Fig. 2b). After 12 days of water deprivation (severe level) the stomatal conductance reached values as low as 0.01 mol H_2_O m^− 2^ s^− 1^ for the susceptible plants (SP80–3280), but no significant difference was found between the two cultivars at that water deficit level. In recovery, the tolerant plants RB92579 showed higher stomatal conductance values (Fig. 2b). Transpiration levels (*E*) of the tolerant and susceptible plants were 60 and 68% lower in moderate water deficit, respectively, compared with those in the irrigated treatment. Upon increased water deprivation (12 days), both cultivars reduced their transpiration rate by 95% in relation to the irrigated treatment. Upon recovery, the increase in *E* was higher in tolerant plants (Fig. 2c).

The susceptible plants showed significantly greater leaf area (*LA*) than RB92579 at the normal water supply condition (Table 1). However, the susceptible plants (SP80–3280) showed superior reduction in *LA* when subjected to water deficit for 3 days and 12 days (about 16 and 38%, respectively). Under these same conditions, the tolerant plants (RB92579) showed a leaf area decrease of approximately 13 and 34%. We did not observe significant differences in leaf (*LDW*) and root dry weight (*RDW*) between the two cultivars in all water deficit regimes (irrigated, moderate and severe levels) (Table 1). However, as observed for *LA*, the RB92579 plants had a smaller reduction in *LDW* and *RDW* after withholding the water supply for 3 and 12 days (19 and 45%, respectively) compared with the susceptible plants under these same water deficit periods (7% for *LDW* and 52% for *RDW*). The susceptible plants (SP80–3280) were characterized by having higher stalk dry weight (*SDW*) under normal water supply condition, which was also reflected in its higher total dry weight (*TDW*) (Table 1). However, when exposed to water deficit, these plants showed larger reduction in *SDW* (36 and 44%, under the moderate and severe water deficit conditions, respectively) compared with the tolerant plants (RB92579). At the end of 12 days of water deprivation (severe level), the total dry weight accumulation was lower in SP80–3280 (42%) than in the tolerant plants (30%) compared to the values in the respective irrigated control plants. No significant differences were observed between the recovery and severe water deficit treatments, showing that 48 h of rehydration was not enough to cause changes in the biomass of the sugarcane plants (Table 1).

**Table 1:** Biomass of tolerant (RB92579) and susceptible (SP80–3280) sugarcane plants grown under irrigated and two water deficit conditions

Cultivar	RB92579			SP80–3280		
Treatment	Irrigated	3 days Moderate	12 days Severe	Irrigated	3 days Moderate	12 days Severe
*LA* (cm^2^)	2658.51 Ba ±114.36	2313.00 Ba ±62.84	1727.82 Bb ±117.22	3316.84 Aa ±110.66	2776.82 Ab ±97.89	2058.72 Ac ±25.65
*LDW* (Kg)	0.12 Aa ±0.001	0.11 Aa ±0.001	0.09 Ab ±0.001	0.12 Aa ±0.003	0.11 Aa ±0.003	0.09 Ab ±0.005
*RDW* (Kg)	0.14 Aa ±0.005	0.12 Aa ±0.002	0.08 Ab ±0.008	0.16 Aa ±0.015	0.12 Ab ±0.004	0.08 Ac ±0.003
*SDW* (Kg)	0.20 Ba ±0.004	0.16 Ab ±0.004	0.14 Ab ±0.004	0.24 Aa ±0.002	0.15 Ab ±0.004	0.13 Ac ±0.004
*TDW* (Kg)	0.46 Ba ±0.009	0.39 Ab ±0.002	0.32 Ac ±0.011	0.52 Aa ±0.012	0.39 Ab ±0.009	0.30 Ac ±0.007

Leaf area (*LA*, cm^2^), leaf dry weight (*LDW*, kg), root dry weight (*RDW*, kg), stalk dry weight (*SDW*, kg) and total dry weight (*TDW*, kg). Different upper case and lower letters indicate statistical differences between cultivars in each treatment and between water deficit treatments (irrigated, 3 and 12 days of water deficit) for each cultivar, respectively, according to Tukey test (*P* < 0.05). Values presented as mean ± SE (*n* = 3)

### Enzymatic activities

The decarboxylating enzymes NADP-ME, NAD-ME and PEPCK, and the aminotransferases AspAT and AlaAT, present only in the NAD-ME and PEPCK subtypes, were assayed.

**Fig. 3:**
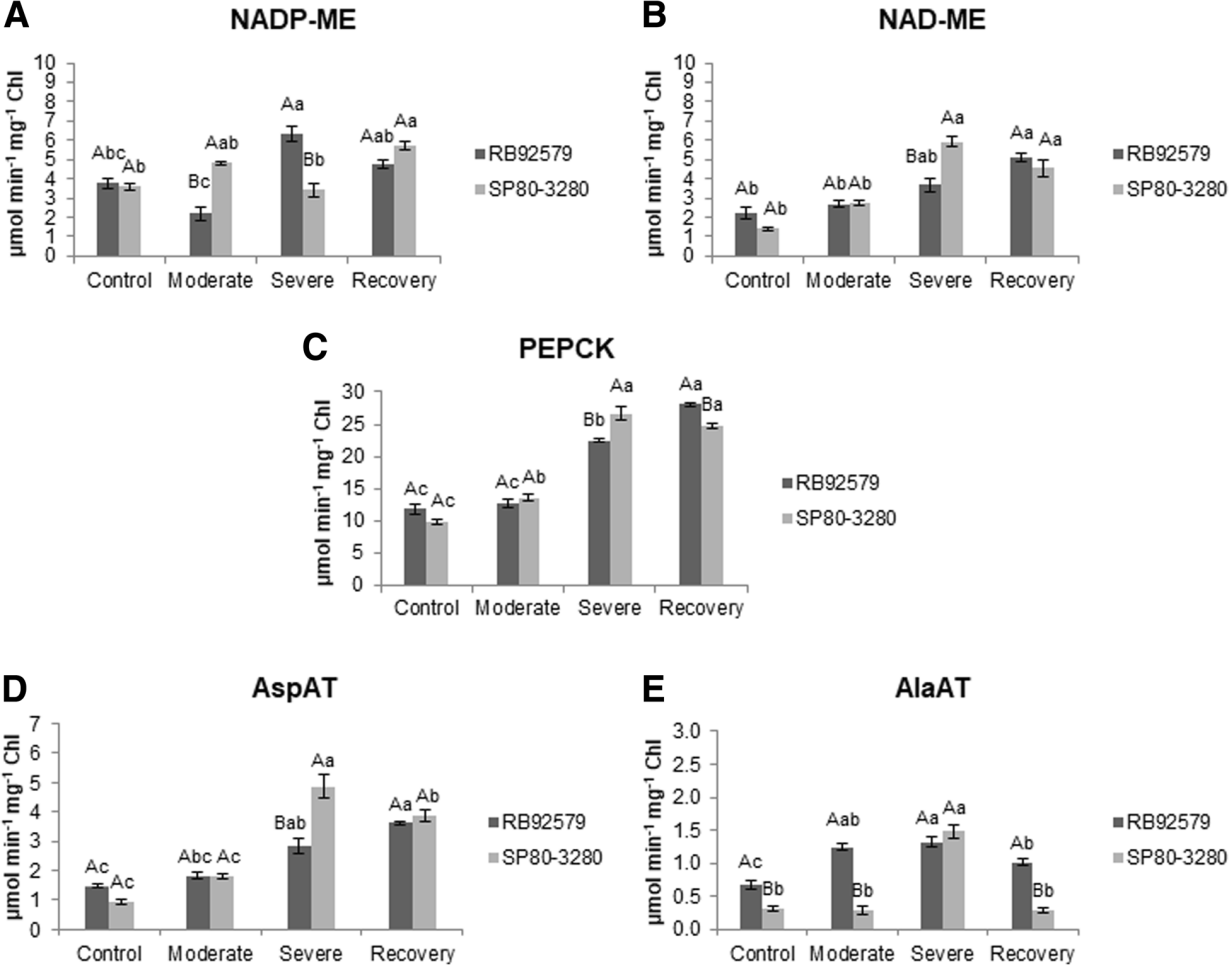
Enzymatic activities in tolerant (RB92579) and susceptible (SP80–3280) sugarcane plant exposed to different water deficit regimes. **a** - NADP-ME; **b** - NAD-ME; **c** - PEPCK; **d** - AspAT; **e** - AlaAT. Different upper case and lower letters indicate statistical differences between cultivars in each treatment and between water deficit treatments (control, moderate, severe and recovery) for each cultivar, respectively, according to Tukey test (*P* < 0.05). Values presented as mean ± SE (*n* = 3) and expressed in μmol min^− 1^ mg^− 1^ Chl

In the tolerant plants (RB92579), the activity of the NADP-ME enzyme was increased in plants under the severe water deficit regime (6.33 μmol min^− 1^ mg^− 1^ Chl). In recovery, the activity of this enzyme declined (25%) to values similar to the control plants. In susceptible plants, the highest activity of this enzyme was observed in the recovery of leaves after the water deficit treatment (5.71 μmol min^− 1^ mg^− 1^ Chl) (Fig. 3a).

Despite a slight increase in the moderate water deficit compared with irrigated plants, the activity of NAD-ME did not differ significantly between the cultivars in these water regimes. On the other hand, the activity of NAD-ME was stimulated in the tolerant plants after rehydration (5.11 μmol min^− 1^ mg^− 1^ Chl), while susceptible plants (SP80–3280) showed the highest activity under severe water deficit (5.92 μmol min^− 1^ mg^− 1^ Chl), maintaining similar values at 48 h after irrigation (Fig. 3b).

Besides showing the highest activity among all the enzymes analyzed under normal water supply, PEPCK also presented a large increase in both cultivars when exposed to deficit conditions. The susceptible plants (SP80–3280) showed a gradual increase in the PEPCK activity until the severe water deficit level, maintaining the high activity state at the recovery treatment (24.78 μmol min^− 1^ mg^− 1^ Chl). Likewise, compared to the irrigated condition, tolerant plants (RB92579) showed a significant increase in PEPCK activity in their leaves at both the severe water deficit (91%) and recovery (138%), with the highest enzyme activity (28.09 μmol min^− 1^ mg^− 1^ Chl) associated with this latter condition (Fig. 3c).

In relation to the aminotransferases, the AspAT activity in the leaves of the tolerant plants showed an increase at each level of water deficit, with the greatest activity at 48 h after rehydration (3.61 μmol min^− 1^ mg^− 1^ Chl). In the susceptible plants (SP80–3280), the activity of this enzyme was higher in the severe water-deprived plants (4.86 μmol min^− 1^ mg^− 1^ Chl), followed by a 20% decline in recovery (Fig. 3d). The activity of AlaAT in the leaves of the tolerant plants (RB92579) was higher than in SP80–3280 plants under irrigated and moderate water deficit regimes, reaching similar values at the severe water restriction level (circa 1.32 μmol min^− 1^ mg^− 1^ Chl). When rehydrated, AlaAT activity in susceptible plants (SP80–3280) returned to the level detected in plants under normal water supply (~ 80% decline), while those of the tolerant plants also decreased, but to a lesser extent (− 23%) (Fig. 3e).

### Analysis of gene expression by RT-qPCR

**Fig. 4:**
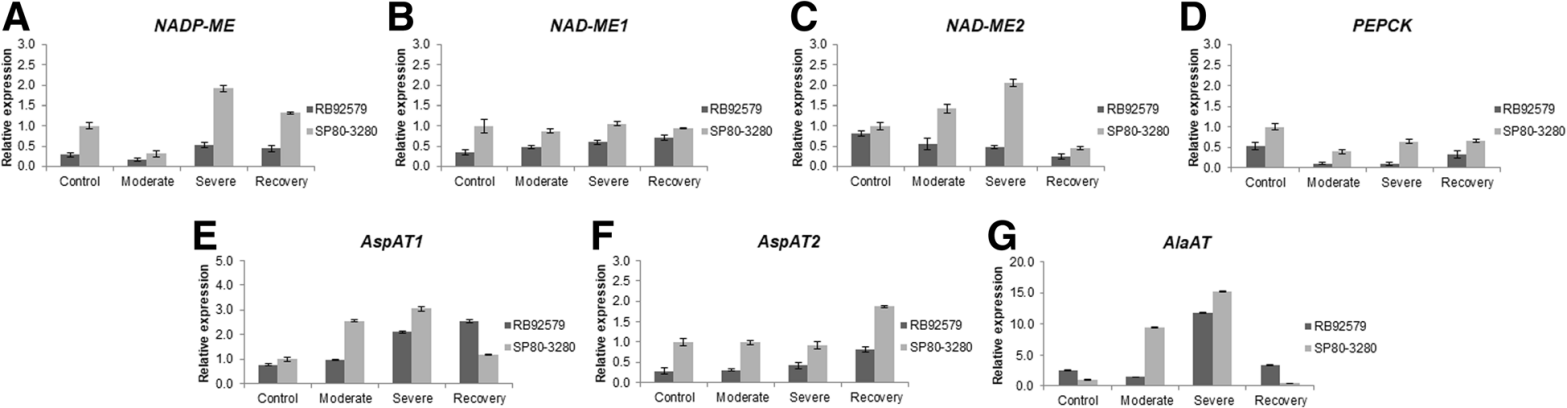
Expression of genes of the C4 pathway of sugarcane plants at different levels of water deficit. **a** - *NADP-ME*; **b** - *NAD-ME1*; **c** - *NAD-ME2;* **d** - *PEPCK;* **e** - *AspAT1*; **f** - *AspAT2;* **g** - *AlaAT*. Values are presented as mean by the E (Efficiency)^-ΔΔCt^ method [65]. Cultivars: RB92579 and SP80–3280; Water deficit treatments: control, moderate, severe and recovery. For the calculations, the *GAPDH* normalizing gene was used and the control treatment of the susceptible plants (SP80–3280) was used. Error bar indicates the ± SD (*n* = 3)

The mRNA profiles of the genes encoding *NADP-ME*, *NAD-ME*, *PEPCK*, *AspAT* and *AlaAT* were significantly altered in sugarcane leaves of both plant types under water deficit (Fig. 4).

*NADP-ME* transcripts were more expressed in leaves of both irrigated and water-deprived plants of the susceptible plants relative to the tolerant one, mainly at the severe water deficit and recovery conditions. The number of transcripts of *NADP-ME* in the tolerant plants (RB92579) had only a small increase under the most severe water deficit level and in the recovery condition when compared to the watered plants (Fig. 4a).

The two *NAD-ME* isoforms (both mitochondrial) were more expressed in the susceptible plants (SP80–3280) in all water regimes. For the *NAD-ME1*, we detected a similar pattern in susceptible plants at all levels of water deficit, whereas an increase in transcript level was associated to the stress intensity in the *NAD-ME2,* with a noticeable drop 48 h after rehydration*.* The tolerant plants (RB92579) exhibited even smaller transcriptional activities for the *NAD-ME1* and *NAD-ME2* isoforms compared with was observed in the susceptible ones (Fig. 4b, c).

In both sugarcane cultivars, *PEPCK* gene was down-regulated in water deprived and rehydrated plants in relation to those grown under normal water supply conditions. The susceptible plants (SP80–3280) expressed higher amounts of transcripts of the *PEPCK* gene than RB92579 in all water regimes (Fig. 4d).

The two *AspAT* gene isoforms responded differently to the water regimes used in this study (Fig. 4e, f). RT-qPCR analysis revealed that there were more transcripts of the *AspAT1* isoform (cytosolic) than *AspAT2* (mitochondrial) in the sugarcane leaves. *AspAT1* showed increased mRNA expression at all water deficit levels in both plant types, but was down-regulated in susceptible plants (SP80–3280) after re-watering. On the other hand, *AspAT2* maintained similar expression levels to the well-watered condition at the moderate and severe drought in both cultivars. The mRNA levels of *AspAT2* increased only 48 h after re-irrigation, and this was more noticeable in the case of the susceptible plants.

Compared with the transcription profiles of the other genes studied here, the *AlaAT* showed the greatest increase in transcripts number after water deprivation. *AlaAT* gene was substantially up-regulated in the sugarcane leaves at the severe water deficit condition in the two plant types (up to 15 fold increase in SP80–3280 under severe level). In the recovery treatment, the mRNA levels of AlaAT severely declined in both susceptible and tolerant plants (Fig. 4g).

## Discussion

Leaf water potential (*ψ*_*w*_) can be considered a good indicator of the water status that maximizes the photosynthetic activity of the sugarcane plant [16, 19]. Water deficit-tolerant plants are able to maintain higher *ψ*_*w*_ values compared to the more susceptible ones under the same water restriction level [20]. Likewise, in this study, the tolerant plants (RB92579) showed a weaker *ψ*_*w*_ reduction when subjected to severe water deficit for a period of twelve days (Fig. 1).

Both cultivars showed a reduction in CO_2_ assimilation rates (*A*) when subjected to water deficit. However, it is interesting to note that tolerant plants also presented a higher assimilation rate compared to susceptible plants under normal water supply conditions (control treatment) (Fig. 2a). In support of these data, it has been previously shown that RB92579 plants achieved higher CO_2_ assimilation, as well as high stalks biomass and sugar content in conditions without water restriction [21]. After recovery, the susceptible plants maintained low CO_2_ assimilation values, indicating that a period of 48 h after rehydration was not sufficient to recover its CO_2_ assimilation capacity, unlike the tolerant plants. This response was observed in previous studies where some sugarcane plants subjected to water deficit showed a minimal increase in the photosynthetic rate following re-watering, demonstrating less ability to return to the original homeostasis [22, 23].

Irrespective of the cultivar, the reduction in *Gs* was more accentuated than the decline in *A*, indicating that the inhibition of photosynthesis by the water deficit was most likely related to stomatal closure in plants under moderate water deficit (Fig. 2a, b). In sugarcane plants under water restriction, the decrease in photosynthesis occurs due to the reduction of stomatal conductance (*Gs*) [24, 25]. As expected, *Gs* values were reduced according to water deficit severity, but there were no significant differences between the plant types in the moderate and severe levels (Fig. 2b), similarly to what was reported in other cultivars [26].

Sugarcane plants subjected to water deficit conditions have significantly lower total dry weight, including dry weight of leaves, roots and stalk, in addition to leaf area compared with non-stressed plants [27]. As expected, the applied water deficit negatively affected the biomass of the two sugarcane plant types used in this study (Table 1). Lower biomass accumulation is associated with foliar gas exchange rates, mainly with reductions in *A* and *Gs* [28, 29]. Interestingly, the less tolerant plants presented higher leaf area and stalk dry weight under irrigated conditions, although its photosynthetic rate was lower than in RB92579, possibly due to counteracting effects linked to compensation mechanisms between leaf area and photosynthetic capacity. It is known that plants with fewer leaves may comparatively show higher photosynthetic rates. Increase of the photosynthetic activity is achieved mainly by enhancing the stomatal conductance but also by an increase of the mesophyll conductance [30, 31]. However, water deprivation resulted in a higher biomass reduction in the susceptible SP80–3280 compared with the tolerant plants.

In sugarcane, it has been reported that the Calvin cycle processes and the C4 pathway are not affected by moderate water stress, but the activities of C4 enzymes are significantly reduced under severe stress [24]. In the present study, the activities of the NADP-ME, NAD-ME, PEPCK, AspAT and AlaAT enzymes showed an increase in both cultivars under severe water deficit, notably the PEPCK enzyme (Fig. 3). A substantial NAD-ME activity was detected in our study, with values similar to those observed for NADP-ME, especially in sugarcane plants under the severe water deficit treatment (Fig. 3a, b). It has been speculated that this enzyme provides the necessary ATP to increase PEPCK activity for sugarcane plants under stress [11, 32]. Western Blot analyses showed that plant species that use predominantly PEPCK have significant levels of NAD-ME [33]. We also detected an increase in the activities of AspAT and AlaAT in both cultivars when exposed to severe water deficit (Fig. 3d, e). In *Z. mays*, the bundle sheath cells showed aspartate decarboxylation capacity and that this process is apparently performed by a sequence of enzymatic activities initiated by AspAT, followed by the activity of the NADP-MDH that generates malate, which is then decarboxylated by NADP-ME in the chloroplast [34]. This aspartate decarboxylation may account for about 20% of the maximum decarboxylation of malate, contributing partially to the total CO_2_ released into bundle sheath cells [7].

Furbank (2011) proposes two hypotheses for the contribution of aspartate to the pool of CO_2_ in the NADP-ME species. The first hypothesis predicts that for PEPCK to be present in NADP-ME species, the oxaloacetate (OAA) produced by aspartate aminotransferase (AspAT) should be directly decarboxylated in the cytosol by PEPCK. This enzyme requires ATP from the chloroplast or mitochondria for decarboxylation to OAA. In the second hypothesis, OAA produced from aspartate is re-reduced to malate in the vascular bundle and then decarboxylated in the chloroplast by NADP-ME [7]. Following the decarboxylation of malate by NADP-ME, the produced NADPH can be used in the Calvin cycle, or it can be spent on reducing of OAA to malate. In this case, pyruvate can return directly to the mesophyll cells or is converted to alanine by the action of the alanine aminotransferase (AlaAT), which in turn is sent to the mesophyll cells. In mesophyll cells, alanine is converted back into pyruvate and, in both cases, there is a need for ATP for pyruvate to be converted to PEP [5, 12]. According to the above considerations, the increase in PEPCK activity could be supported by the increment in the activities of several enzymes: NAD-ME to provide ATP necessary for this decarboxylase, AspAT to form the oxaloacetate (OAA) and AlaAT for the production of alanine through pyruvate, which is, in turn, directed to mesophyll cells [5]. This hypothesis was reinforced in our study, as we detected an increased activity of these enzymes when the plants were exposed to the water deficit, particularly in the susceptible plants (SP80–3280) under severe water deprivation (Fig. 3). Collectively our data favor the second hypothesis for the contribution of aspartate to the pool of CO_2_ in the NADP-ME species [5]. The increased number of transcripts and enzyme activity (Figs. 3, 4) of AspAT to form OAA, PEPCK for decarboxylation of OAA forming PEP and AlaAT for production of alanine through pyruvate support this view. The flexible use of distinct C4 pathways may potentially allow the maintenance of greater photosynthetic efficiency under different environmental conditions, such as those applied in this study. This is because the use of only NADP-ME requires more energy for the conversion of pyruvate into PEP in mesophyll cells, in addition to NADPH for conversion of OAA to malate. With the use of PEPCK, PEP has the ability to return directly to the mesophyll cells and is already used in the carboxylation, without any additional energy expenditure and without the need for the PPDK enzyme.

The rise in PEPCK activity can be modulated by various environmental factors [5, 11]. Specifically in sugarcane, shading caused increases in the decarboxylation by PEPCK, which shows that this enzyme is the main decarboxylase under low light conditions [11]. These authors showed that *PEPCK* decarboxylation uses less quanta *per* CO_2_ fixed than *NADP-ME*, thus the increase in *PEPCK* activity advantageous contribute to maintaining or even increasing quantum efficiency of CO_2_ assimilation under limiting light. Our data showed that by increasing water deficit level, the activity of the enzymes important for the reactions involved in PEPCK decarboxylation also increased (Fig. 3).

Given the evidence that the regulation of enzymes associated with C4 photosynthesis is affected by water restriction, we analyzed the transcriptional level of the genes encoding these enzymes [18]. The increase in the number of transcripts of the *NADP-ME* gene under severe water deficit in the susceptible plants (SP80–3280) can be explained by the fact that under these stress conditions the stomatal conductance is reduced and the amount of CO_2_ entering in the mesophyll cells also decreases. In this case, as the CO_2_ input is lower, there would be an increase in the expression of this enzyme to supply the CO_2_ required for the Calvin cycle [35] (Fig. 4a). It has been reported that the *PEPCK* and *AspAT* transcripts are not significantly enriched under water deficit sugarcane plants at stalk elongation stage, whereas *AlaAT* was highly induced [36], which also occurred in our work. However, in contrast to that study, we observed that one of the isoforms encoding aspartate aminotransferase (*AspAT1*) exhibited high transcriptional activity under severe water restriction (nine days after irrigation suspension) (Fig. 4e). The high number of transcripts of the *AspAT2* and *AlaAT* genes may be associated with the high activity of PEPCK observed in our work (Fig. 3c, 4f and g). In using this second decarboxylase, other enzymes involved in this pathway are more necessary to keep the decarboxylation mechanism working efficiently. This has already been demonstrated in *Z. mays* and *Setaria viridis*, species belonging to the NADP-ME subtype, where the *AspAT* and *AlaAT* genes were highly expressed in mesophyll and bundle sheath cells [37, 38].

Although the PEPCK showed high activity in sugarcane plants under severe water deprivation (Fig. 3c), the expression of the gene encoding this enzyme did not follow the same pattern and reduced the number of transcripts when the two cultivars were exposed to water deficit (Fig. 4d). The relationship between enzymatic activities and transcription levels of genes may be the result of various factors, such as circadian rhythms, transcript stability, transcription factors, enhancer activities, post-transcriptional and post-transductional regulation pathways, substrate availability and the involvement of different isoforms in biological processes that occur at diverse temporal and spatial levels [39]. It is known that the activity of PEPCK is regulated by various factors, such as light, biotic and abiotic stress [40] and the phosphorylation of PEPCK has been observed in some C4 leaves [41]. Therefore, it is possible that the upregulation of the PEPCK decarboxylation activity in the sugarcane plants under water deprivation observed here might be mediated by via phosphorylation–dephosphorylation reactions is tempting to speculate that the upregulation of the PEPCK activity in the sugarcane plants under water deficiency observed here does not occur at the transcriptional level, but rather via modulation of phosphorylation–dephosphorylation reactions. The purification of this enzyme and cloning of the complete cDNA sequence of the *PPCK* gene may be relevant for the identification of specific phosphorylation sites and their functional significance in sugarcane under conditions of water deficiency.

When sugarcane plants were subjected to water deficit, there was a large increase in the mRNA expression of one isoform of the NAD^+^ dependent malic enzyme (*NAD-ME2*) in the susceptible plants (SP80–3280), especially under the severe water deficit. However, the expression of this gene was still lower than that observed for *NADP-ME* under normal water supply conditions (control). In this case, six more cycles of amplification (*C*_*q*_ - quantification cycle) were required for the detection of the *NAD-ME2* gene in comparison with the *NADP-ME* gene in both cultivars (Additional file 2: Figure S2). Even taking into account the difference in efficiency of the primers, these data show that the *NADP-ME* gene is more expressed (about 50-fold) than *NAD-ME2* in plants under the non-stressing conditions (Fig. 4a, c). Also, the *C*_*q*_ values for the *PEPCK* mRNAs in plants under normal water supply were similar to those detected for *NADP-ME* in both cultivars (Additional file 2: Figure S2). The high expression of the *PEPCK* gene was also observed in bundle sheath cells of maize leaves, a species classified as NADP-ME subtype [42]. Therefore, the preferred expression of *NAD-ME* and *NADP-ME* genes may not be exclusively correlated with their corresponding subtype.

As mentioned above, increased gene expression and activity of AspAT and AlaAT enzymes, which are not characteristic to the metabolic pathways used in the classical NADP-ME subtype [2, 4], were detected in sugarcane under water deficit conditions. In agreement with that, in a transcriptome study with this species using the SAGE technique, no *NADP-MDH* transcripts were detected, while a high mRNA levels of *PEPCK* and *AspAT* genes were identified [10]. The observed lack (or low levels) of *NADP-MDH* transcription comparatively to the higher expression of *PEPCK* and *AspAT* suggests that the PEPCK decarboxylation pathway seems to function in sugarcane leaves, both under conditions irrigated as well as under water deficit conditions. Our data support recent research that indicates the occurrence of mixed decarboxylation C4 pathways in various plants previously considered to belong exclusively to a single subtype of C4 photosynthetic mechanism [12].

Added to the difficulty of classifying sugarcane in C4 subtypes only by the predominance of the corresponding enzymatic activity, is the remarkable fact that sugarcane is the only named NADP-ME species in which the presence of mestome sheath cells were reported to date [43, 44]. Plants of the NADP-ME subtype do not possess a mestome sheath, which is a layer of suberized non-photosynthetic cells located between the bundle sheath cells and the vascular bundles (Additional file 3: Figure S3). Sugarcane is the only interesting exception because the presence of mestome around its vascular bundles has been clearly detected [43–46]. In addition, it is worth mentioning that changes in chloroplast arrangement of bundle sheath cells, from centrifugal (feature found in the classical NADP-ME species) to evenly distributed (typical of the PEPCK subtype) were observed in sugarcane plants grown under shading possibly to increase CO_2_ diffusion between the sheath and mesophyll cells, reducing CO_2_ loss [11]. These authors suggested that such a rearrangement is important to maintain the efficiency of CO_2_ assimilation in suboptimal light conditions, which also may be the case during water shortage conditions.

Finally, although ^13^C flux measurements in a wider panel of contrasting cultivars are required to obtain direct evidence linking PEPCK to drought tolerance, the great increase in the use of the PEPCK pathway under water deficit conditions shows that this aspect needs to be further explored.

## Conclusion

The enzymatic activity and gene expression analyses indicate that sugarcane uses the PEPCK pathway as a decarboxylation mechanism, in addition to the NADP-ME pathway. Under conditions of water deficit, a greater increase in the use of the PEPCK pathway was detected, particularly in the tolerant cultivar, which recommends additional investigations to examine the complementary use of the PEPCK decarboxylation pathway as a response and adaptation of other sugarcane plants to water deprivation.

## Methods

### Plant material and water deficit treatment

Sugarcane plants were produced from single node stalk segments (circa 7 cm in length) from cv. RB92578, tolerant to water deficit [47] and cv. SP80–3280, susceptible to water deficit [48]. Plants of a similar size (pruned to one stalk per plant) of each cultivar were grown in plastic pots (15 L) filled with 13 kg of eutrophic red-yellow argisoil [49] under greenhouse conditions. Soil fertilization was performed according to chemical analysis by adding 5 g of 20–00-20 (NPK) per pot at 135 DAE (days after emergence) and again at 165 DAE [50]. All plants were watered daily to drip point until the beginning of the drought treatment.

The water deficit treatment started 210 days after of shoot emergence at the stalk elongation phase. Plant water status was evaluated daily through the leaf water potential (*ψ*_*w*_) measured with a pressure chamber (PMS 1000, PMS Instruments, USA) at midday in the third fully-expanded leaf. The treatments consisted of different levels of water deficit: control (normal conditions of water supply, *ψ*_*w*_ = − 0.4 to − 0.6 MPa), moderate level (*ψ*_*w*_ = − 1.5 to − 1.8 MPa) three days after the onset of the water deprivation, severe level (*ψ*_*w*_ = below − 2.0 MPa) twelve days after the onset of the water deprivation and re-watered plants (48 h after rehydration, *ψ*_*w*_ = − 0.6 to − 1.0 MPa). In the control treatment, the pots were maintained at 100% field capacity, while for the water deficit treatment the soil water content was adjusted to 30% of field water holding capacity by daily watering according to evapotranspirated water estimated by weighing the pots for twelve days. The two cultivars reached the established water deficit levels at the same time: the moderate condition occurred three days after the imposition of the water deficit, while the severe level occurred nine days after that date, totaling twelve days of water shortage.

### Gas exchange measurements

Leaf gas exchange analysis was performed in the + 1 leaf (first leaf fully expanded from top to bottom [51]), when the plants reached the established water deficit levels (3 and 12 days after water withholding, and 48 h after rehydratation), always at midday. The environmental conditions during the analysis were PAR (photosynthetic active radiation) 800 μmol m^− 2^ s^− 1^, air temperature between 28 and 29 °C, average humidity 67 ± 3.3%, leaf temperature 31.7 ± 0.6 °C and VpdL 2.4 ± 0.6 kPa. The measurements were made in the + 1 leaf under irradiation of 1600 μmol m^− 2^ s^− 1^ and CO_2_ concentration of 400 ppm^− 1^. Leaf tissue was placed in the leaf chamber and briefly acclimated for 5 min prior to the measurements until the stabilization of the readings. CO_2_ assimilation (*A*), stomatal conductance (*Gs*) and transpiration (*E*) were quantified using an infrared gas analyzer (model Li-6400XTR, LI-COR, Lincoln, USA). The analyzed leaves were harvested, immediately frozen in liquid N and stored in ultrafreezer (model MDF-U33 V-PE VIP ULT, Panasonic, Loughborough, UK) at − 80 °C until the enzymatic and RT-qPCR analyses. After recovery, the plants were collected for the biomass analysis.

### Enzymatic assays

Extracts were obtained by grinding 20 mg of frozen leaf samples, removed from the middle third of the + 1 leaf, in 1 mL of ice-cold extraction medium. For the enzymes NADP-ME, NAD-ME and PEPCK, the buffer contained 50 mM Bicine-KOH (pH 8.0), 1 mM EDTA, 5% (*w*/*v*) PVP 25000, 6% (w/v) PEG 4000, 10 mM DTT, 50 mM 2-mercaptoethanol and 1% (*v*/v) protease inhibitor cocktail [14]. For AspAT and AlaAT, the buffer consisted of 10% (v/v) glycerol, 0.25% (w/v) BSA, 0.1% (v/v) Triton X-100, 50 mM Hepes-KOH (pH 7.5), 10 mM MgCl_2_, 1 mM EDTA, 1 mM EGTA, 1% (v/v) protease inhibitor cocktail and 1 mM DTT [52].

The maximal activities of the NADP-ME (EC 1.1.1.40), NAD-ME (EC 1.1.1.39) and PEPCK (EC 4.1.1.49) enzymes were quantified [14]. For NADP-ME the reaction mixture (1.5 mL) contained 50 mM Hepes-KOH (pH 8.0), 10 mM MgCl_2_, 0.5 mM NADP^+^, 5 mM L-malate and 40 μL of leaf extract. For NAD-ME, a solution (1.5 mL) containing 50 mM Hepes-KOH (pH 7.2), 4 mM MnCl_2_, 0.1 mM CoA, 4 mM NAD^+^, 5 mM L-malate and 40 mL of leaf extract was used for the quantification. In both analyzes, the extract was incubated with all reagents, except the substrate, for 3 min at 25 °C, and the reaction was started with the addition of L-malate. The PEPCK was determined using a 1.5 mL solution containing 100 mM Hepes-KOH (pH 7.0), 100 mM KCl, 90 mM KHCO_3_, 5 mM MgCl_2_, 2 mM MnCl_2_, 1 mM ADP, 0.2 mM NADH, 12 U malate dehydrogenase, 5 mM PEP and 40 μL of leaf extract. The reaction was started with the addition of PEP. In these three procedures, the absorbance was monitored by spectrophotometry (model UV-M51, Bel, Rio de Janeiro, Brazil) for 1 min at a wavelength of 340 nm.

The enzymatic activities of AspAT (EC 2.6.1.1) [53] and AlaAT (EC 2.6.1.2) [54] also were determined. For AspAT, reactions were performed in a solution containing 50 mM Tris-HCl (pH 7.8), 50 mM L-aspartate, 10 mM 2-oxoglutarate, 0.07 mM pyridoxal phosphate, 0.1 mM NADH, 2 U malate dehydrogenase and 40 μL of leaf extract in a final volume of 1.5 mL. For AlaAT, a solution (1.5 mL) containing 10 mM L-alanine, 5 mM 2-oxoglutarate, 0.1 mM NADH, 50 mM Tris-HCl (pH 7.5), 5 U lactate dehydrogenase and 40 μL of leaf extract. In both analyses, the leaf extract was incubated with all reagents except 2-oxoglutarate for 3 min at 25 °C and the reaction was started with the addition of 2-oxoglutarate. The absorbance change was monitored by spectrophotometry at 340 nm for 3 min immediately after the addition of the substrate in two replicates for each of three biological replicates per treatment.

The enzymatic activities were based on the total chlorophyll content. For this, chlorophyll was determined from 20 mg of leaf tissue maintained in 80% acetone solution for 24 h at 4 °C and protected from light. The determination was performed by spectrophotometry at 645 nm and 663 nm [55].

### Biomass

At the end of the experiment, the plants subjected to 3 days (moderate level) and 12 days (severe level) of water deficit were removed from the pots and divided into stalks plus leaves and roots for the evaluation of the biomass. The total leaf area was measured with a leaf area meter (LI-3000A, LI-COR, Lincoln, USA). The dry weight of leaves and roots were recorded and averaged after drying the samples in an air drier at 60 °C until obtaining a constant weight using a precision balance. The dry weight of the stalk also was evaluated [56].

### Selection, search and analysis of candidate gene sequences

We evaluated the levels of mRNAs encoding the following enzymes: 1, *NADP-ME* (NADP-malic enzyme); 2, *NAD-ME* (NAD-malic enzyme); 3, *PEPCK* (phosphoenolpyruvate carboxykinase); 4, *AspAT* (aspartate aminotransferase); 5, *AlaAT* (alanine aminotransferase). These genes were chosen for analysis primarily due to their role in the three subtypes of C4 photosynthesis [57, 58]. The identification of sugarcane transcripts related to the synthesis of the above-mentioned enzymes was done by identity to the corresponding sorghum (*Sorghum bicolor*) orthologs. The sequences from the coding region of each sorghum gene were used as queries against sugarcane ESTs from cv. SP80–3280 using the BLAST tools (https://blast.ncbi.nlm.nih.gov/Blast.cgi). The sugarcane ESTs were acquired from (1) a set of complete coding sequences obtained using 454 sequencing technology [59] and (2) the “Gene Indices Database”, available at ftp://occams.dfci.harvard.edu/pub/bio/tgi/data/Saccharum_officinarum/).

The selected sugarcane transcripts were then compared to the sorghum genome sequences available on GenBank using BLASTX option to ensure that the transcript was the best hit against its sorghum ortholog (accession, e-value, score). Additionally, the sorghum sequences were aligned with the contigs corresponding to the sugarcane genes through the ClustalW multiple alignment algorithm (https://www.genome.jp/tools-bin/clustalw) to assess the pattern of conservation between the sequences (Table 2). To ensure a thorough analysis, MultiLoc2 [60] and TargetP [61] were used to predict the subcellular localization of the proteins of each enzyme.

**Table 2:** A summary of genes description and primers sequences used for RT-qPCR

Gene	Description	ESTs *Saccharum* spp.	Locus *Sorghum bicolor*	Primer forward (5′-3′)	Primer reverse (5′-3′)	Amplicon (bp)	Efficiency (%)
*NADP-ME*	NADP-malic enzyme	comp88554_c0_seq1^1^	Sb03g003230	GTGAGGCCTGCCAGAAGTAT	CTAGGACCTTCCCCTTGTCC	84	99
*NAD-ME1*	NAD-malic enzyme	comp79553_c1_seq2^1^	Sb01g017790	TACAGGGGACAGCTGGAGTT	CTCCCACAACGACGATCTTT	102	95.5
*NAD-ME2*	NAD-malic enzyme	comp83394_c0_seq4^1^	Sb02g033920	CAGCAGTTCCCTGAACATCA	CGCTGGCCTAATGTTATCGT	118	92.5
*PEPCK*	Phosphoenol-pyruvate carboxykinase	comp81929_c0_seq5^1^	Sb01g040720	CTGTCGCAGGAGAAAGAACC	CAGATTTGTCGGCGTAGTCA	112	100
*AspAT1*	Aspartate aminotransferase	comp82581_c0_seq1^1^	Sb03g035220	GCACAGTCCTCATGCTCAAA	AATAGCGAGCAAGTGGCATT	97	93
*AspAT2*	Aspartate aminotransferase	comp83051_c1_seq5^1^	Sb04g036060	CGCGTTTAACAAAGCAACAG	CCGAGAGAGACTGAATTGTAGC	94	97
*AlaAT*	Alanine aminotransferase	TC113519^2^	Sb01g023750	TGCCACAGAAAGCAATTGAG	GGACCACGACAATTCCAGTT	99	98.5
*GAPDH*	Glyceraldehyde-3-phosphate dehydrogenase	SCBFFL4116A05.g^3^	–	CACGGCCACTGGAAGCATCCTCAG	TCCTCAGGGTTCCTGATGCC	101	97.5

^1^[59]

^2^(ESTs obtained in ftp://occams.dfci.harvard.edu/pub/bio/tgi/data/.)

^3^(GenBank EST)

### Primer design

Primers were designed using the program Primer3Plus [62] according to parameters established to obtain amplicons of 80 to 120 bp with a Tm of 57 °C to 60 °C, length 20 to 23 bp and GC 40 to 80% (Table 2). Sequences of the genes with more than one isoform (*NAD-ME* and *AspAT*) were aligned through the ClustalW program to identify conserved regions and to design primers specific for each isoform. To avoid non-specific annealing to other transcripts, all primer pairs were challenged against *Saccharum* spp. sequences deposited on GenBank using Primer-Blast (https://www.ncbi.nlm.nih.gov/tools/primer-blast/).

### RNA extraction and cDNA synthesis

Total RNA was extracted from 100 mg of leaf tissue removed from the middle third of the + 1 leaf using the PureLink Plant™ RNA Reagent (Invitrogen, Carlsbad, CA, USA) following the manufacturer’s instructions. Contaminating DNA was removed using RNase-free DNase I (Turbo DNase, Ambion, Austin, TX). The yield of total RNA was assessed spectrophotometrically at 260 nm UV, its relative purity estimated by the absorbance ratio at 260/280 nm and the integrity examined on 1.2% (*w*/*v*) ethidium bromide-stained agarose gels. The absence of genomic DNA contamination was confirmed by the lack of genomic DNA amplification of the endogenous glyceraldehyde-3-phosphate dehydrogenase (*GAPDH*) gene by PCR.

cDNA was synthesized using SuperScript IV reverse transcriptase (Invitrogen, Carlsbad, CA, USA) from 2 μg total RNA according to the manufacturer’s instructions. Specific products and amplicon length of all primer pairs were verified by PCR using a pool of all cDNA samples solutions under the following conditions: initial denaturation at 95 °C for 5 min, followed by 40 cycles of 95 °C for 1 min, 57 °C for 30 s, 72 °C for 30 s, and a final extension step of 72 °C for 2 min. The amplified products were subjected to electrophoresis on 1.5% (w/v) agarose gel to confirm the product size.

### RT-qPCR

The RT-qPCR reactions were performed in a StepOnePlus™ Real-Time PCR System (Applied Biosystems), using the SYBR Green PCR Master Mix (Applied Biosystems). Each reaction consisted of 1 μl of cDNA sample (1:5 diluted), 5 μl of SYBR Green PCR master mix, and 0.4 μl of each primer (5 μM) and nuclease-free water to a total volume of 10 μl. Thermal conditions were 95 °C for 2 min, followed by 40 cycles of 95 °C for 30 s and 60 °C for 30 s. All reactions were performed in triplicate for each of the three biological replicates, following the minimum information for publication of qPCR experiments (MIQE) [63]. Melting curves were analyzed to verify the presence of nonspecific products and primers were used only in the case of a single peak. The mean amplification efficiency of each primer pair was calculated by the LinRegPCR program [64] using a pool of cDNA derived from all samples (Table 2).

The relative quantification of the expression of each gene was calculated using the (1 + E)^-ΔΔCt^ method [65]. In all analyses, the transcript levels of the target genes were normalized against the endogenous reference gene glyceraldehyde-3-phosphate dehydrogenase (*GAPDH*) [66]. The relative mRNA levels of each target gene in SP80–3280 plants grown under normal water supply conditions were used as calibrator.

### Statistical analysis

The experiment was carried out in 2 × 4 factorial arrangement (cultivar x water regime), in a completely randomized design with three replicates within each water regime. Each replicate was composed of one plant per pot, totaling 24 plants. Data from the gas exchange measurements, biomass and enzymatic activities were subjected to analysis of variance (ANOVA), when significant, the means were compared by the Tukey test at 5% of probability using the statistical program SISVAR version 5.3 [67].

## Additional files

Additional file 1: **Figure S1.** Three biochemical subtypes of C4 photosynthesis. 1. Carbonic anhydrase (AC); 2. Phosphoenolpyruvate carboxylase (PEPC); 3. NADP-malate dehydrogenase (NADP-MDH); 4. NADP-malic enzyme (NADP-ME); 5. Pyruvate orthophosphate dikinase (PPDK); 6. Aspartate aminotransferase (AspAT); 7. NAD-malic enzyme (NAD-ME); 8. Alanine aminotransferase (AlaAT); 9. Phosphoenolpyruvate carboxykinase (PEPCK). Metabolites: PEP - Phosphoenolpyruvate; OAA - Oxaloacetate, Asp - Aspartate; Ala - Alanine; Pyr - Pyruvate; Mal - Malate. Chl - Chloroplast (green); Mito - mitochondria (red). CC - Calvin cycle. (PDF 31 kb)

Additional file 2: **Figure S2.** Cycles of quantification (*C*_*q*_) of genes of the C4 pathway for the tolerant (RB92579) and susceptible (SP80–3280) sugarcane plants under the different water deficit regimes (12 replicates). The Boxplot chart shows the median values as rows in the box. The lower and upper boxes indicate quartiles 1 and 3, respectively. The bars represent the upper and lower limits. (PDF 242 kb)

Additional file 3: **Figure S3.** Differences in leaf anatomy of C4 NADP-ME and PEPCK species. Abbreviations: MC - mesophyll cells; BS - bundle sheath; VB - vascular bundle; MS - mestome sheath. A. NADP-ME species show suberized BS, absence of MS and centrifugally arranged chloroplasts. B. PEPCK species have BS and MS suberized and chloroplasts show variable placement. (PDF 283 kb)

## Abbreviations

*A*: Net CO_2_ assimilation
AlaAT: Alanine aminotransferase
AspAT: Aspartate aminotransferase
*C*_*q*_: Quantification cycle
*E*: transpiration
*GAPDH*: Glyceraldehyde-3-phosphate dehydrogenase
*Gs*: Stomatal conductance
*LA*: Leaf area
*LDW*: Leaf dry weight
NAD-ME: NAD-malic enzyme
NADP-MDH: NADP-dependent malate dehydrogenase
NADP-ME: NADP-malic enzyme
OAA: Oxaloacetate
PEP: Phosphoenolpyruvate
PEPC: Phosphoenolpyruvate carboxylase
PEPCK: phosphoenolpyruvate carboxykinase
PPDK: Orthophosphate pyruvate dikinase
*RDW*: Root dry weight
SAGE: Serial analysis of gene expression
*SDW*: Stalk dry weight
*TDW*: Total dry weight
*ψ*_*w*_: water potential

## References

[1] Furbank RT, Hatch MD (1987). Mechanism of C4 photosynthesis: the size and composition of the inorganic carbon pool in bundle-sheath cells. Plant Physiol.

[2] Maier A, Zell MB, Maurino VG (2011). Malate decarboxylases: evolution and roles of NAD(P)-ME isoforms in species performing C_4_ and C_3_ photosynthesis. J Exp Bot.

[3] Bowyer JR, Leegood RC, Dey PM, Harbone JB (1997). Photosynthesis. Plant biochemistry.

[4] Hatch MD, Kagawa T, Craig S (1975). Subdivision of C_4_-pathway species based on differing C_4_ acid decarboxylating systems and ultrastructural features. Aust J Plant Physiol.

[5] Furbank RT (2011). Evolution of the C4 photosynthetic mechanism: are there really three C4 acid decarboxylation types?. J Exp Bot.

[6] Hatch MD (1971). C_4_-pathway of photosynthesis. Evidence for an intermediate pool of carbon dioxide and identity of donor C_4_-dicarboxylic acid. Biochem J.

[7] Chapman KSR, Hatch MD (1981). Aspartate decarboxylation in bundle sheath cells of *Zea mays* and its possible contribution to C4 photosynthesis. Aust J Plant Physiol.

[8] Furumoto T, Hata S, Izui K (2000). Isolation and characterization of cDNAs for differentially accumulated transcripts between mesophyll cells and bundle sheath strands of maize leaves. Plant Cell Physiol.

[9] Walker RP, Chen ZH, Acheson RM, Leegood RC (2002). Effects of phosphorylation on phosphoenolpyruvate carboxykinase from the C4 plant Guinea grass. Plant Physiol.

[10] Calsa T, Figueira A (2007). Serial analysis of gene expression in sugarcane (*Saccharum* spp.) leaves revealed alternative C4 metabolism and putative antisense transcripts. Plant Mol Biol.

[11] Sales CRG, Ribeiro RV, Hayashi AH, Marchiori PER, Silva KI, Martins MO (2018). Flexibility of C4 decarboxylation and photosynthetic plasticity in sugarcane plants under shading. Environ Exp Bot.

[12] Wang Y, Bräutigam A, Weber AP, Zhu XG (2014). Three distinct biochemical subtypes of C4 photosynthesis? A modelling analysis. J Exp Bot.

[13] Bellasio C (2017). A generalized stoichiometric model of C_3_, C_2_, C_2_+C_4_, and C_4_ photosynthetic metabolism. J Exp Bot.

[14] Carmo-Silva AE, Silva AB, Keys AJ, Parry MA, Arrabaça MC (2008). The activities of PEP carboxylase and the C4 acid decarboxylases are little changed by drought stress in three C4 grasses of different subtypes. Photosynth Res.

[15] Bräutigam A, Schliesky S, Külahoglu C, Osborne CP, Weber AP (2014). Towards an integrative model of C4 photosynthetic subtypes: insights from comparative transcriptome analysis of NAD-ME, NADP-ME, and PEP-CK C4 species. J Exp Bot.

[16] Inman-Bamber NG, Smith DM (2005). Water relations in sugarcane and response to water deficits. Field Crop Res.

[17] Bellasio C, Quirk J, Beerling DJ (2018). Stomatal and non-stomatal limitations in savanna trees and C_4_ grasses grown at low, ambient and high atmospheric CO_2_. Plant Sci.

[18] McCormick AJ, Cramer MD, Watt DA (2008). Changes in photosynthetic rates and gene expression of leaves during a source–sink perturbation in sugarcane. Ann Bot.

[19] Endres L, Silva JV, Ferreira VM, Barbosa GVS (2010). Photosynthesis and water relations in Brazilian sugarcane. Open Agr J.

[20] Pincelli RP (2010). Tolerância à deficiência hídrica em cultivares de cana-de-açúcar avaliada por meio de variáveis morfofisiológicas.

[21] Ferreira RA, de Souza JL, Lyra GB, Teodoro I, dos Santos MA, Porfirio ACS (2012). Crescimento e fotossíntese de cana-de-açúcar em função de variáveis biométricas e meteorológicas. Rev Bras Eng Agr Amb.

[22] da Graça JP, Rodrigues FA, Farias JRB, de Oliveira MCN, Hoffmann-Campo CB, Zingaretti SM (2010). Physiological parameters in sugarcane cultivars submitted to water deficit. Braz J Plant Physiol.

[23] Souza GM, Oliveira RF, Machado EC (2004). Temporal dynamics of stomatal conductance of plants under water deficit: can homeostasis be improved by more complex dynamics?. Braz Arch Biol Techn.

[24] Du YC, Nose A, Wasano K, Uchida Y (1998). Responses to water stress of enzyme activities and metabolite levels in relation to sucrose and starch synthesis, the Calvin cycle and the C_4_ pathway in sugarcane (*Saccharum* spp.) leaves. Aust J Plant Physiol.

[25] Chaves MM, dos Santos TP, Souza C, Pereira JS (2007). Deficit irrigation in grapevine improves water-use efficiency while controlling vigour and production quality. Ann Appl Biol.

[26] Gonçalves ER, Ferreira VM, Silva JV, Endres L, Barbosa TP, Duarte WG (2010). Trocas gasosas e fluorescência da clorofila a em variedades de cana-de-açúcar submetidas à deficiência hídrica. Rev Bras Eng Agr Amb..

[27] Barbosa AM, Guidorizi KA, Catuchi TA, Marques TA, Ribeiro RV, Souza GM (2015). Biomass and bioenergy partitioning of sugarcane plants under water deficit. Acta Physiol Plant.

[28] Zhao D, Glaz B, Comstock JC (2013). Sugarcane leaf photosynthesis and growth characters during development of water-deficit stress. Crop Sci.

[29] Flexas J, Bota J, Galmés J, Medrano H, Ribas-Carbó M (2006). Keeping a positive carbon balance under adverse conditions: responses of photosynthesis and respiration to water stress. Physiol Plant.

[30] Morrison KD, Reekie EG (1995). Pattern of defoliation and its effect on photosynthetic capacity in *Oenothera biennis*. J Ecol.

[31] Meinzer F (2002). Co-ordination of vapour and liquid phase water transport properties in plants. Plant Cell Environ.

[32] Kanai R, Edwards GE, Sage R, Russel M (1998). The biochemistry of C_4_ photosynthesis. C_4_ plant biology.

[33] Voznesenskaya EV, Franceschi VR, Chuong SD, Edwards GE (2006). Functional characterization of phosphoenolpyruvate carboxykinase-type C4 leaf anatomy: Immuno-, cytochemical and ultrastructural analyses. Ann Bot.

[34] Pick TR, Bräutigam A, Schlüter U, Denton AK, Colmsee C, Scholz U (2011). Systems analysis of a maize leaf developmental gradient redefines the current C4 model and provides candidates for regulation. Plant Cell.

[35] Doubnerová V, Ryslavá H (2011). What can enzymes of C4 photosynthesis do for C3 plants under stress?. Plant Sci.

[36] Li C, Nong Q, Solanki MK, Liang Q, Xie J, Liu X (2016). Differential expression profiles and pathways of genes in sugarcane leaf at elongation stage in response to drought stress. Sci Rep.

[37] Chang YM, Liu WY, Shih ACC, Shen MN, Lu CH, Lu MYJ (2012). Characterizing regulatory and functional differentiation between maize mesophyll and bundle sheath cells by transcriptomic analysis. Plant Physiol.

[38] John CR, Smith-Unna RD, Woodfield H, Covshoff S, Hibberd JM (2014). Evolutionary convergence of cell-specific gene expression in independent lineages of C4 grasses. Plant Physiol.

[39] de Carvalho K, de Campos MK, Domingues DS, Pereira LF, Vieira LGE (2013). The accumulation of endogenous proline induces changes in gene expression of several antioxidant enzymes in leaves of transgenic *Swingle citrumelo*. Mol Biol Rep.

[40] Shen Z, Dong XM, Gao ZF, Chao Q, Wang BC (2017). Phylogenic and phosphorylation regulation difference of phosphoenolpyruvate carboxykinase of C3 and C4 plants. J Plant Physiol.

[41] Walker RP, Leegood RC (1996). Phosphorylation of phosphoenolpyruvate carboxykinase in plants: studies in plants with C4 photosynthesis and Crassulacean acid metabolism and in germinating seeds. Biochem J.

[42] Tausta SL, Li P, Si Y, Gandotra N, Liu P, Sun Q (2014). Developmental dynamics of Kranz cell transcriptional specificity in maize leaf reveals early onset of C4-related processes. J Exp Bot.

[43] Robinson-Beers K, Evert RF (1991). Fine structure of plasmodesmata in mature leaves of sugarcane. Planta.

[44] Robinson-Beers K, Evert RF (1991). Ultrastructure of and plasmodesmatal frequency in mature leaves of sugarcane. Planta.

[45] Elahi NN, Ashraf M (2001). *Study of various sized leaf vascular* bundles and surrounding tissues of six sugarcane varieties. Pak J Biol Sci.

[46] Joarder N, Roy AK, Sima SN, Parvin K (2010). Leaf blade and midrib anatomy of two sugarcane cultivars of Bangladesh. J Biol Sci.

[47] Barbosa GVS, Sousa AJR, Rocha AMC, dos Santos AVP, Ribeiro CAG, Barreto EJS (2003). Três novas variedades RB de cana-de-açúcar.

[48] News I (2004). Características agronômicas da cultivar SP80–3280.

[49] dos Santos HG, Jacomine PKT, dos Anjos LHC, de Oliveira VA, Lumbreras JF, Coelho MR (2008). Sistema brasileiro de classificação de solos.

[50] Malavolta E, Vitti GC, Oliveira SA (1997). Avaliação do estado nutricional das plantas: princípios e aplicações.

[51] Purdy LH, Dean JL (1981). A system for record data about sugarcane rust/host interactions. Sugarcane Pathol New.

[52] Gibon Y, Blaesing OE, Hannemann J, Carillo P, Höhne M, Hendriks JHM (2004). A robot-based platform to measure multiple enzyme activities in *Arabidopsis* using a set of cycling assays: comparison of changes in enzyme activities and transcript levels during diurnal cycles and in prolonged darkness. Plant Cell.

[53] Yagi T, Kagamiyama H, Nozaki M, Soda K (1985). Glutamate-aspartate transaminase from microorganisms. Method Enzymol.

[54] Good AG, Muench DG (1992). Purification and characterization of an aerobically induced alanine aminotransferase from barley roots. Plant Physiol.

[55] Arnon DI (1949). Copper enzymes in isolated chloroplasts polyphenoloxidase in *Beta vulgaris*. Plant Physiol.

[56] Fernandes AC (2003). Cálculos na agroindústria canavieira.

[57] Rocha FR, Papini-Terzi FS, Nishiyama MY, Vêncio RZN, Vicentini R, Duarte RDC (2007). Signal transduction-related responses to phytohormones and environmental challenges in sugarcane. BMC Genomics.

[58] Iskandar HM, Casu RE, Fletcher AT, Schmidt S, Xu J, Maclean DJ (2011). Identification of drought-response genes and a study of their expression during sucrose accumulation and water deficit in sugarcane culms. BMC Plant Biol.

[59] Nishiyama MY, Ferreira SS, Tang PZ, Becker S, Pörtner-Taliana A, Souza GM (2014). Full-length enriched cDNA libraries and ORFeome analysis of sugarcane hybrid and ancestor genotypes. PLoS One.

[60] Blum T, Briesemeister S, Kohlbacher O (2009). MultiLoc2: integrating phylogeny and gene ontology terms improves subcellular protein localization prediction. BMC Bioinformatics.

[61] Emanuelsson O, Nielsen H, Brunak S, von Heijne G (2000). Predicting subcellular localization of proteins based on their N-terminal amino acid sequence. J Mol Biol.

[62] Untergasser A, Nijveen H, Rao X, Bisseling T, Geurts R, Leunissen JA (2007). Primer3Plus, an enhanced web interface to Primer3. Nucleic Acids Res.

[63] Bustin SA, Benes V, Garson JA, Hellemans J, Huggett J, Kubista M (2009). The MIQE guidelines: minimum information for publication of quantitative real-time PCR experiments. Clin Chem.

[64] Ramakers C, Ruijter JM, Deprez RH, Moorman AF (2003). Assumption-free analysis of quantitative real-time polymerase chain reaction (PCR) data. Neurosci Lett.

[65] Livak KJ, Schmittgen TD (2001). Analysis of relative gene expression data using real-time quantitative PCR and the 2^-∆∆Ct^ method. Methods.

[66] Guo J, Ling H, Wu Q, Xu L, Que Y (2014). The choice of reference genes for assessing gene expression in sugarcane under salinity and drought stresses. Sci Rep.

[67] Ferreira DF (2010). SISVAR - Sistema de análise de variância. Versão 5.3.

