# Supplementary figures and images for: Decarboxylation mechanisms of C4 photosynthesis in Saccharum spp.: increased PEPCK activity under water-limiting conditions
Source: BMC Plant Biol. 2019 Apr 16;19:144. doi: 10.1186/s12870-019-1745-7 (PMC6469216; doi:10.1186/s12870-019-1745-7)

## NADP-ME

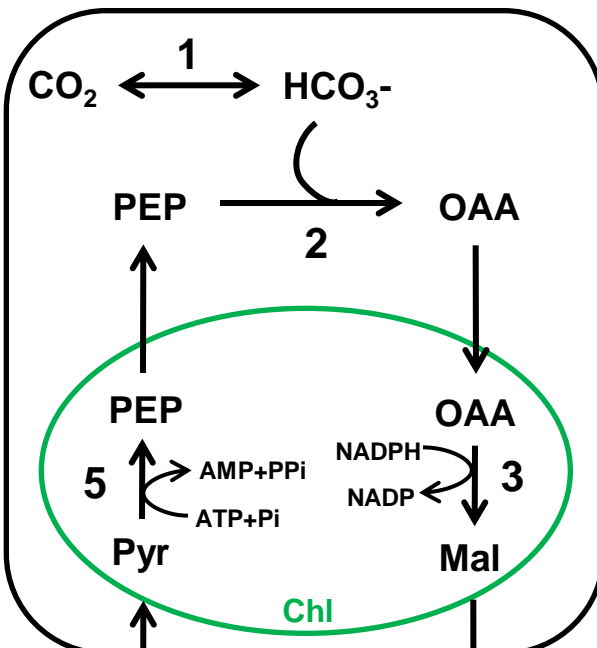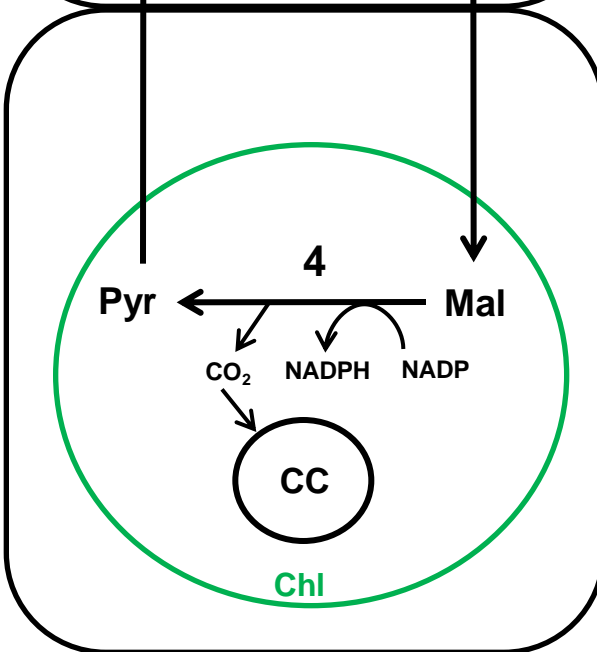

## NAD-ME

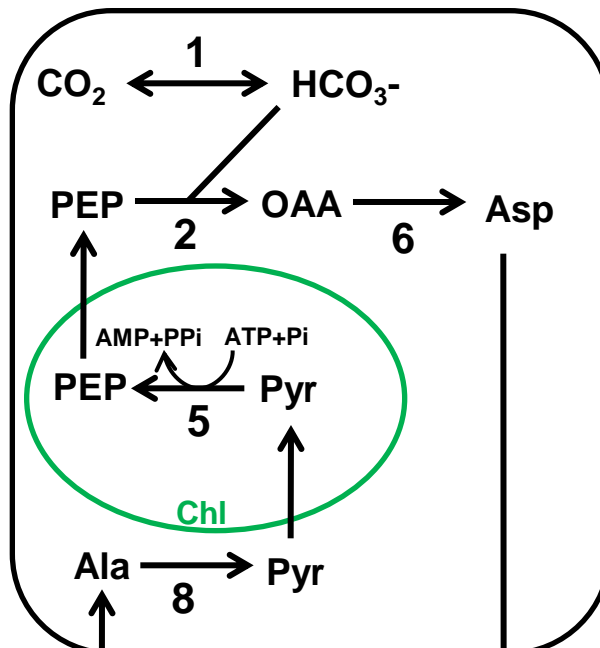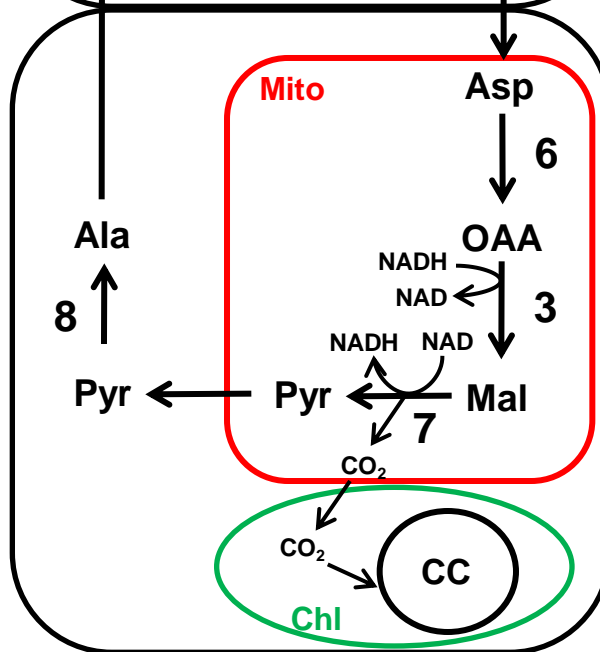

## PEPCK

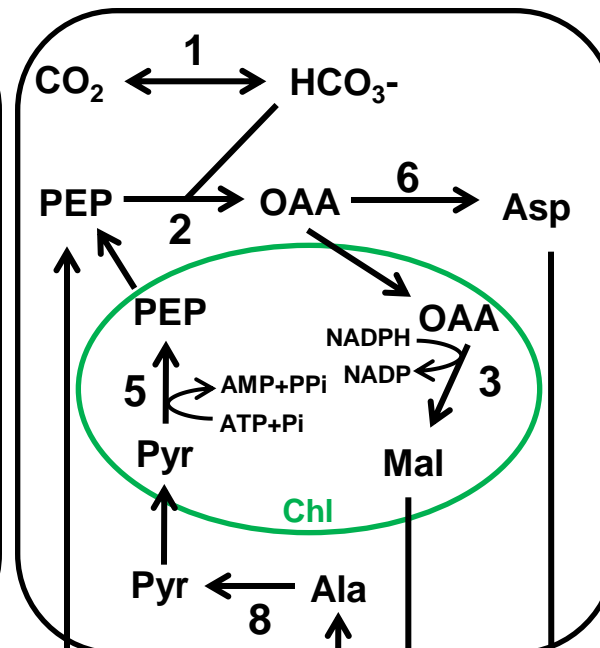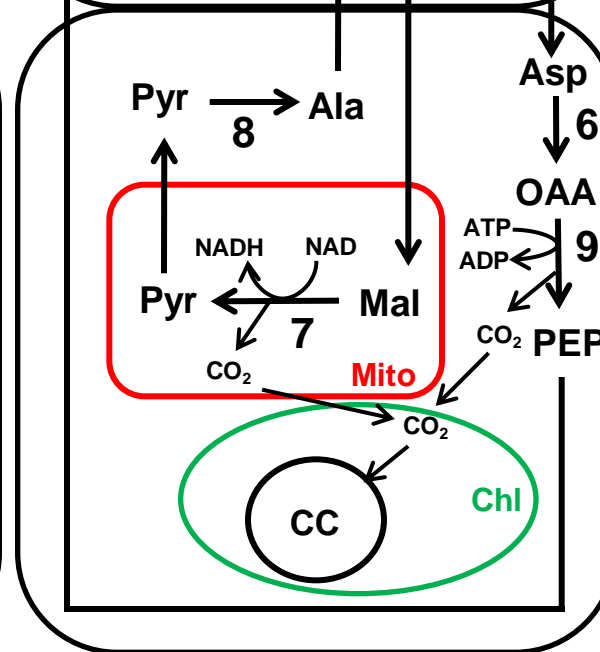

Mesophyll cell

Bundle sheath cell

Supplement: Supplementary file 1 — Figure S1. Three biochemical subtypes of C4 photosynthesis. 1. Carbonic anhydrase (AC); 2. Phosphoenolpyruvate carboxylase (PEPC); 3. NADP-malate dehydrogenase (NADP-MDH); 4. NADP-malic enzyme (NADP-ME); 5. Pyruvate orthophosphate dikinase (PPDK); 6. Aspartate aminotransferase (AspAT); 7. NAD-malic enzyme (NAD-ME); 8. Alanine aminotransferase (AlaAT); 9. Phosphoenolpyruvate carboxykinase (PEPCK). Metabolites: PEP - Phosphoenolpyruvate; OAA - Oxaloacetate, Asp - Aspartate; Ala - Alanine; Pyr - Pyruvate; Mal - Malate. Chl - Chloroplast (green); Mito - mitochondria (red). CC - Calvin cycle. (PDF 31 kb) [file 12870_2019_1745_MOESM1_ESM.pdf]

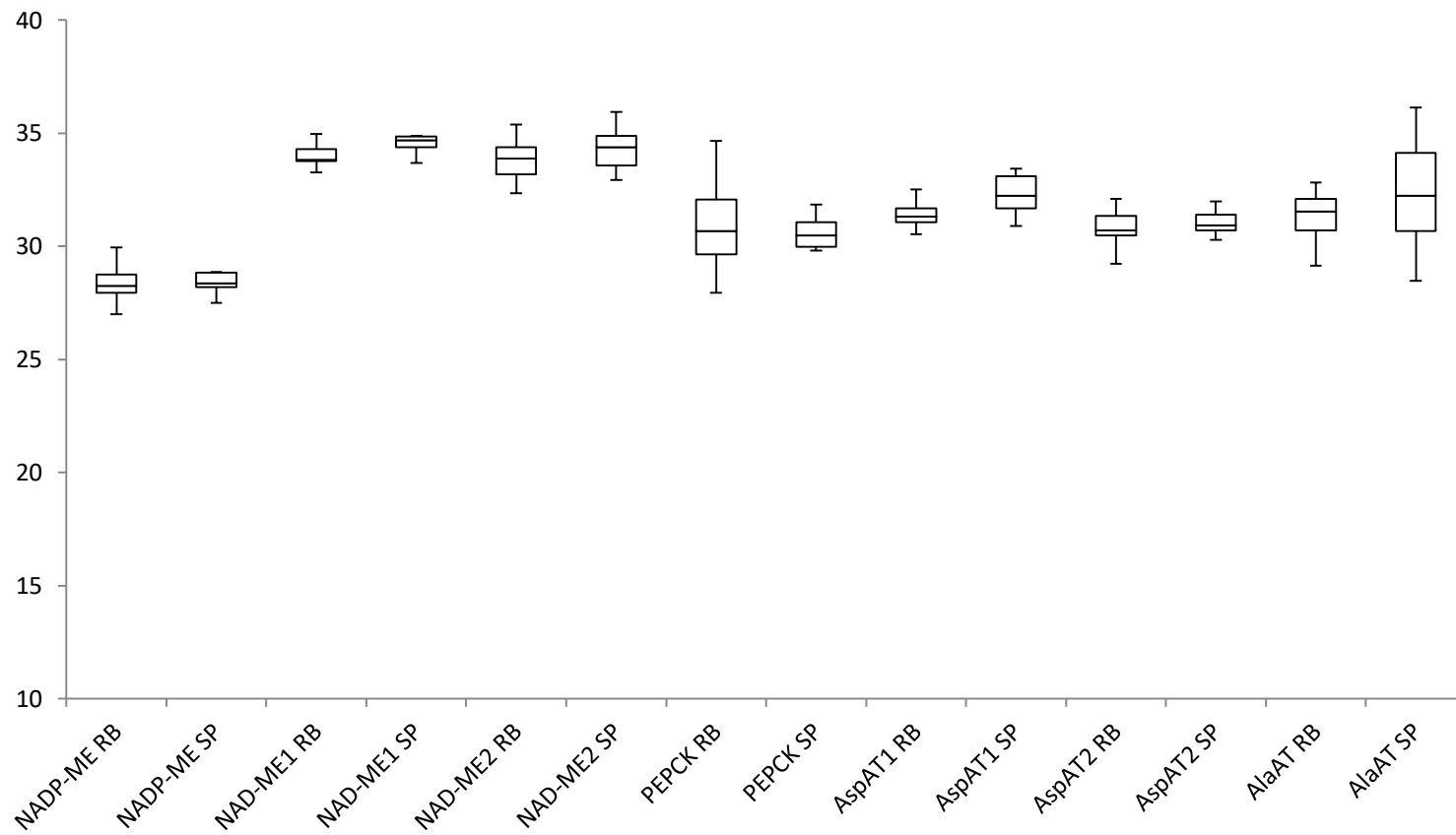

Supplement: Supplementary file 2 — Figure S2. Cycles of quantification (Cq) of genes of the C4 pathway for the tolerant (RB92579) and susceptible (SP80–3280) sugarcane plants under the different water deficit regimes (12 replicates). The Boxplot chart shows the median values as rows in the box. The lower and upper boxes indicate quartiles 1 and 3, respectively. The bars represent the upper and lower limits. (PDF 242 kb) [file 12870_2019_1745_MOESM2_ESM.pdf]
