# Supplementary material for: Decarboxylation mechanisms of C4 photosynthesis in Saccharum spp.: increased PEPCK activity under water-limiting conditions
Source: BMC Plant Biol. 2019 Apr 16;19:144. doi: 10.1186/s12870-019-1745-7 (PMC6469216; doi:10.1186/s12870-019-1745-7)
Supplement: Supplementary file 3 — Figure S3. Differences in leaf anatomy of C4 NADP-ME and PEPCK species. Abbreviations: MC - mesophyll cells; BS - bundle sheath; VB - vascular bundle; MS - mestome sheath. A. NADP-ME species show suberized BS, absence of MS and centrifugally arranged chloroplasts. B. PEPCK species have BS and MS suberized and chloroplasts show variable placement. (PDF 283 kb) [file 12870_2019_1745_MOESM3_ESM.pdf]

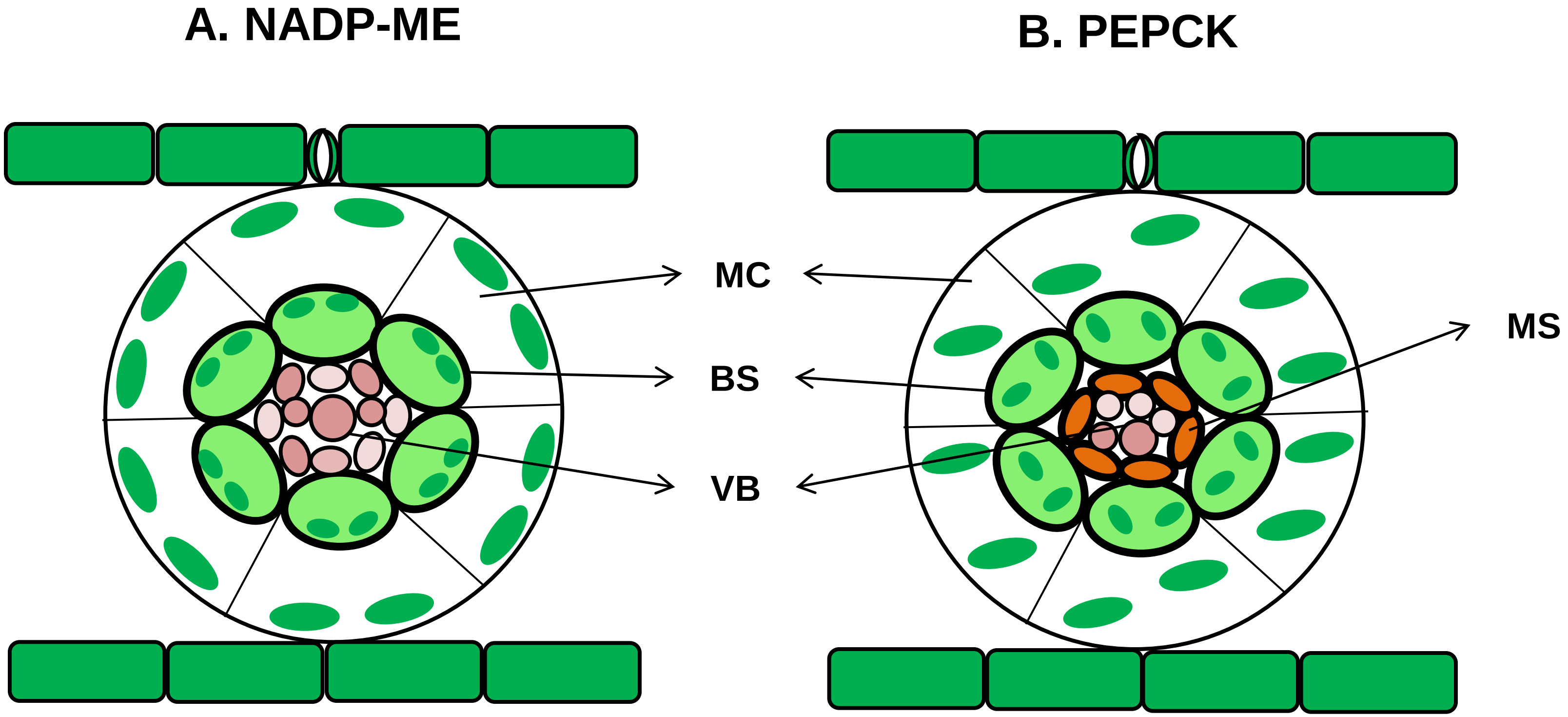
A. NADP-ME
B. PEPCK
MC
BS
VB
MS

Figure S3 – Differences in leaf anatomy of C4 NADP-ME and PEPCK species. Abbreviations: MC - mesophyll cells; BS - bundle sheath; VB - vascular bundle; MS - mestome sheath. A. NADP-ME species show suberized BS, absence of MS and centrifugally arranged chloroplasts. B. PEPCK species have BS and MS suberized (long black lines) and chloroplasts show variable placement. Adapted from Mertz and Brutnell (2014).
